# Prognostic significance of *SNCA* and its methylation in bladder cancer

**DOI:** 10.1186/s12885-022-09411-9

**Published:** 2022-03-26

**Authors:** Zhengcun Wu, Chengxing Xia, Chao Zhang, Delin Yang, Kaili Ma

**Affiliations:** 1 Institute of Medical Biology, Chinese Academy of Medical Sciences and Peking Union Medical College, Kunming, 650118 China; 2 Department of Urology, The Second Affiliated Hospital of Kunming Medical University, Kunming, 650101 China; 3 Oncology Department, The First People’s hospital of Qujing, Qujin, 655000 China; 4 Medical Primate Research Center & Neuroscience Center, Chinese Academy of Medical Sciences, Beijing, 100005 China; 5 Yunnan Key Laboratory of Vaccine Research Development on Severe Infectious Diseases, Kunming, 650118 China

## Abstract

**Background:**

The epidemiological investigation of different cancer types in the global population has reported a decreased risk of bladder cancer (BLCA) in Parkinson’s diseases (PD). *SNCA* a critical gene in PD pathology have been reported involved in tumorigenesis recently. However, the role of *SNCA* in BLCA remains unclear. This study aimed to explore the potential value of *SNCA* as a prognostic diagnostic molecular biomarker in BLCA.

**Methods:**

In this study, we explored the expression pattern, prognostic value and promoter methylation level of *SNCA* in BLCA by GEPIA2, UALCAN, TCGA, GENT2, GEO and c-BioPortal database. Then, we used LinkedOmics database to obtain the co-expression genes of *SNCA* for further study by WGCNA. We further investigated the correlations between *SNCA* expression and six main types of immune cell infiltrations and immune signatures by TIMER. Finally, BLCA cell lines treated with 5-Aza-CdR were used to explore the correlation between increased methylation and downregulated mRNA expression.

**Results:**

*SNCA* was downregulated in tumor tissues in TCGA-BLCA, GENT2 and GEO, which was validated in our cohort by qRT-PCR and immunohistochemistry. *SNCA* was confirmed as an independent predictor of poor overall survival (OS). LinkedOmics analysis suggested that *SNCA* regulates cell adhesion molecules, cytokine–cytokine receptor interaction, and complement and coagulation cascades. Twenty-two co-expression gene modules were constructed by WGCNA, and most of them were significantly associated with OS and disease-free survival (DFS). Six key genes (*CNTN1*, *DACT3*, *MYLK1*, *PDE2A*, *RBM24*, and *ST6GALNAC3*) screened also significantly correlated with prognosis. There were significant correlations between *SNCA* expression and immune infiltrations, especially T cell, suggesting that immune infiltration was one of the reasons for the influence of *SNCA* on prognosis in BLCA. Analysis by ULACAN and c-BioPortal showed that the promoter methylation of *SNCA* negatively correlated with its mRNA level. Furthermore, BLCA cell treatment with 5-Aza-CdR revealed that *SNCA* expression levels were upregulated with decreased methylation.

**Conclusion:**

Our research showed that *SNCA* was downregulated in BLCA and negatively correlation with DNA methylation. High *SNCA* expression was confirmed as an independent risk for prognosis. *SNCA* probably plays an important role in the infiltration of immune cells, especially with T cells. Thus, *SNCA* may be a promising prognostic biomarker in BLCA patients.

**Supplementary Information:**

The online version contains supplementary material available at 10.1186/s12885-022-09411-9.

## Introduction

*SNCA*, located on chromosome 4q22.1, encodes a 140-amino acid protein, namely alpha synuclein (α-Syn). α-Syn is a presynaptic neuronal protein, which plays a crucial role in the etiology of Parkinson’s disease (PD) and other synucleinopathies [1]. Converging evidence from various in vitro and in vivo studies has suggested that α-Syn misfolding and aggregation is major pathogenic event in PD. Mutations in *SNCA* were the first identified genetic causes of PD [2]. Besides the key role in synapse function, α-Syn has many other biological functions, such as apoptosis induction, oxidative stress elevation, regulation of calcium and mitochondrial homeostasis, and cell cycle aberrations [3].

Recent studies have demonstrated the potential role of *SNCA* in the pathological processes underlying human cancers, including ovarian and breast cancer [4], colorectal tumor [5], melanoma [6], brain cancer [7] and lung adenocarcinoma [8]. *SNCA* methylation levels are significantly upregulated in colon cancer patients, and thus can be used as a biomarker for noninvasive detection [9]. Through microarray analysis of drug-resistant microRNAs, Zou et al. demonstrated that downregulation of *SNCA* was significantly associated with multidrug resistance in ovarian cancer [10]. Other studies have shown that *SNCA* is involved in the progression of breast cancer [11]. Li et al. have reported that *SNCA* can be used as a new diagnostic marker for medulloblastoma, and proved that *SNCA* may inhibit tumor growth by inducing apoptosis via activating the Akt/mTOR pathway [12]. In medulloblastomas, the immune response of *SNCA* can be observed [13]. *SNCA* promoter hypermethylation has been reported as an early diagnostic indicator of Hodgkin’s lymphoma [14]. Previously, A53T α-Syn transgenic mice were generated to evaluate the *SNCA* on tumorigenesis. The results revealed that melanoma and breast cancer were accelerated, but no effect on lung cancer was observed, indicating that *SNCA* may selectively accelerate cellular mechanisms leading to cancer [15].

Interestingly, an epidemiological investigation of different cancer types in the global population has reported a decreased risk of bladder cancer (BLCA) in PD patients (OR/RR = 0.62; 95% CI, 0.42–0.91; I^2^ = 88.3%, *P* < 0.001) [16]. However, few studies have discussed reasons for this inverse association. BLCA is the second most common genitourinary malignancy, with over 550,000 cases diagnosed worldwide in 2020 [17]. Despite neoadjuvant and adjuvant chemotherapy, the outcomes of metastatic BLCA are poor [18]. To provide a highly accurate prediction of a patient’s survival and/or response to individualized treatment therapy, identification of new biomarkers of BLCA is needed. In addition, the lack of markers that are specific for tumor type or disease stage represents a critical gap in the current understanding and treatment of BLCA. We speculated that *SNCA* is key molecular link between PD and BLCA, and may serve as a novel biomarker for BLCA.

In this study, we first explored the expression of *SNCA* and its impact on prognosis in patients with BLCA based on The Cancer Genome Atlas (TCGA) and various public databases. Using the LinkedOmics database, we obtained the genes co-expressed with *SNCA* and used WGCNA to further study their potential biological function. In addition, due to the key role of immune microenvironment in tumor development, we evaluated the potential correlation between *SNCA* and immune infiltration through TIMER database. Finally, analysis was performed for the methylation level of *SNCA* promoter with its mRNA and protein levels. The expression of *SNCA* in BLCA cell lines was studied by treatment with 5-Aza-CdR, a demethylating agent. Our results could potentially reveal *SNCA* as a new prognostic marker for BLCA.

## Materials and methods

### Download data from TCGA, GEO and GENT2 database

Level 3 gene expression profile (FPKM, level 3 data) and clinical data for BLCA patients were obtained from the TCGA data portal (https://tcga-data.nci.nih.gov/tcga/), then we downloaded RNA expression and clinical data for BLCA from GEO database, including GSE13507, GSE3167, GSE32894 and GSE32548. Gene Expression database of Normal and Tumor tissues 2 (GENT2) is an updated version of GENT, which has provided a user-friendly search platform for gene expression patterns across different normal and tumor tissues compiled from public gene expression data sets [19]. We obtained expression of *SNCA* in GPL570 and GPL96 between normal and tumor tissues.

### Gene expression profiling interactive analysis (GEPIA) database

The GEPIA is an interactive web that provides experimental biologists and clinicians with a convenient tool to explore TCGA and GTEx datasets. GEPIA2 (http://gepia2.cancer-pku.cn/) is an updated and enhanced version, which provides insights with higher resolution and more functionalities. It features 198,619 isoforms and 84 cancer subtypes [20]. GEPIA2 was used to analyze *SNCA* expression between normal and tumor, and the survival map of OS.

### UALCAN database analysis

UALCAN (http://ualcan.path.uab.edu) is a database that uses TCGA level 3 RNA sequencing and clinical data from 31 cancer types. It can analyze relative expression of a query gene(s) across tumor and normal samples, as well as in various tumor subgroups based on individual cancer stages, tumor grade, race, body weight, or other clinic-pathological features [21]. This resource serves as a platform for in silico validation of target genes and for identifying tumor subgroup-specific candidate biomarkers. It is built on PERL-CGI and can be used to assess the methylation level of different genes. In this study, the *SNCA* expression and promotor methylation profile was tested in the UALCAN database based on patients’ individual cancer stages, age, gender, race, and smoking status.

### C-BioPortal database analysis

The cBio Cancer Genomics Portal (http://cbioportal.org) has multidimensional cancer genomics data sets [22]. We calculated the correlation between gene expression and methylation of *SNCA* in BLCA was analyzed using the c-BioPortal tool. The scatter plot displays expression and methylation level per sample in *SNCA.*

### Functional enrichment and pathway enrichment analysis

KEGG (Kyoto Encyclopedia of Gene and Genomes) (www.kegg.jp/kegg/kegg1.html) is a knowledge for systematic analysis of gene functions, linking genomic information with higher order functional information. The higher order functional information is stored in the PATHWAY database [23]. In this study, Gene Ontology (GO) and KEGG pathway enrichment analyses were performed for genes within the key module based on “clusterProfiler” in R software, an open-source programming environment, and have been released under Artistic License 2.0 within Bio-conductor project [24]. Gene sets with a *q* value less than 0.05 were considered significantly enriched.

### Survival analysis

The Kaplan–Meier survival curves (http://cran.r-project.org/web/packages/survival/index.html) were created using “survival” R packages. For survival analysis, BLCA patients were categorized into the high group and the low group, according to the *SNCA* mRNA Optimal grouping threshold, which was estimated by R package “survminer”. The Log-Rank test was used to estimate the overall survival (OS) and recurrence-free survival (RFS) differences between groups with different expression levels of *SNCA*. *P* -value < 0.05 was selected as threshold.

### LinkedOmics database analysis

The LinkedOmics database (http://www.linkedomics.org/login.php) is a multi-omics database that contains multi-omics data and clinical data for 32 cancer types and a total of 11,158 patients from the TCGA project [25]. *SNCA* co-expression was analyzed statistically using Pearson correlation coefficient and presented in volcano plots and heat maps. Function modules to GO biological process (GO_BP) and KEGG enrichment were set by gene set enrichment analysis (GSEA). The rank criterion was false-discovery rate (FDR) < 0.05, and 1000 simulations were performed.

### Establishment of weighted co-expression network (WGCNA)

Genes co-expressed with *SNCA* from LinkedOmics database were selected based on a common threshold (FDR < 0.01). All selected genes were used to construct a weighted correlation network using the R package “WGCNA” [26]. The R function pick SoftThreshold was used to decide the soft thresholding power β (power = 6). The hierarchical clustering and the dynamic tree cut function embedded in WGCNA were used to detect the functional modules. Then, the relationships between all detected modules and clinical traits were evaluated based on the correlation score of the eigengene for each module and the measured clinical traits. The hub genes for each module were chosen according to the module membership (MM > 0.8) and the difference between intramodular and intermodular connectivity (Kdiff > 0). The corresponding top five hub genes for each module as well as the eigengene for each module were extracted for survival analysis. The *p* values were calculated using log-rank test. Finally, the brown module in the WGCNA analysis significantly related to BLCA OS and DFS was further analyzed for GO and KEGG enrichment.

### TIMER database analysis

TIMER (https://cistrome.shinyapps.io/timer/) is a comprehensive resource for the analysis of immune infiltrates and the differences in gene expression in different tumors from TCGA [27]. We analyzed *SNCA* expression in BLCA and the correlation of *SNCA* expression with the abundance of immune infiltrates, including B cells, CD4+ T cells, CD8+ T cells, neutrophils, macrophages, and dendritic cells, as well as the tumor purity. Multivariate Cox analysis was used to evaluate how *SNCA* and these six types of immune cells together affected survival prognosis.

### Analysis of immune signatures

ESTIMATE is a database used for the single-sample gene-set enrichment analysis (ssGSEA) sore to quantify the enrichment level of immune signatures in a tumor. The tumor purity was estimated using ESTIMETE as previously described [28]. Gene signatures of chemokine, receptor, major histocompatibility complex (MHC), immunoinhibitory, immunostimulatory, and 28 tumor-infiltrating lymphocytes (TILs) were analyzed by TISIDB, which is a central portal for tumor and immune system interactions [29]. The correlations between *SNCA* and these gene signatures were calculated via the “Correction” module of TIMER with tumor purity-corrected partial Spearman’s correlation coefficients.

### Clinical samples

Tumor and adjacent non-tumorous tissues of 20 patients with BLCA, who underwent surgical treatment at the Department of Urology, the Second Affiliated Hospital of Kunming Medical University from January 2019 to February 2021 were collected (Table 1). The study was approved by the Academic Committee of the Second Affiliated Hospital of Kunming Medical University, and it was conducted in accordance with the principles expressed in the Declaration of Helsinki. All 20 patients with BLCA signed written informed consent and did not receive other special treatment before surgery. All the data sets were retrieved from the published literature, confirming that all written informed consents were obtained.

**Table 1:** The clinical characteristics information for 20 patients with BLCA in this study

Variables	Incidence or mean value (%)
Age (yrs)	67.8 ± 13.52
Gender, n (%)	
Male	16 (80.0)
Female	4 (20.0)
Grade, n (%)	
G1	4 (20.0)
G2	11 (55.0)
G3	5 (25.0)
Stage, n(%)	
NMIBC	
Ta	0 (0.0)
T1	4 (20.0)
MIBC	
T2	12 (60.0)
T3	3 (15.0)
T4	1 (5.0)

*NMIBC* non-muscle invasive bladder cancer, *MIBC* muscle invasive bladder cancer

### Quantitative real-time polymerase chain reaction (qRT-PCR)

Total RNA was isolated from tissue samples using TRIzol reagent (Sigma-Aldrich, USA). The concentration and quality of the RNA were determined by NanoPhotometer (IMPLEN, Germany). Next, cDNA was generated from 2 μg total RNA using the iScript™ cDNA Synthesis Kit (Promega, USA) in accordance with the manufacturer’s instructions. To detect mRNA levels of genes, qRT-PCR was performed using Eastep qPCR Master Mix (Promega, USA) and a CFX96 Real-Time PCR Detection System (Bio-Rad, USA). The primers’ sequences are listed in Supplementary Table S1. The comparative threshold (Ct) was used to calculate the amount of cDNA, normalized by the Ct of *GAPDH*. The relative gene expression levels were presented as relative quantification values, which were calculated using the 2 − ΔΔCt method.

### Screening and verification of key genes

We defined the top five genes in the 22 module networks as hub genes. A total of 20 genes among the hub genes that significantly correlated with OS and DFS were selected for further studies. The univariate Cox analysis was further conducted to screen potential prognostic genes for OS. Genes with a *p* value less than 0.01 were regarded as key genes. The expression of key genes from BLCA patients was analyzed by qRT-PCR.

### Immunohistochemistry (IHC)

IHC was performed as described previously [30]. After antigen retrieval with sodium citrate buffer in a microwave oven and 15-min incubation with 3% H_2_O_2_ to block endogenous peroxidase activity, the tissue microarrays were incubated with anti-*SNCA* antibody (Abcam, USA) overnight at 4 °C and then treated with secondary antibodies (Abcam, USA) for 1 h. Finally, they were stained with diaminobenzidine (DAB) until brown granules appeared. The slides were observed using a laser multicolor fluorescence scanning imager (GE, USA). Image-Pro Plus 6.0 soft was used to analyze the number of positive cells and total cells in tissue microarray. The final protein level were measured by histochemistry score (H-score), a semi quantitative method for IHC of tissues.

### Cell culture and treatment

The human BLCA cell lines T24 (RRID: CVCL_0554), EJ (RRID: CVCL_2893), 5637(RRID: CVCL_0126), and J82 (RRID: CVCL_0359) were purchased from ATCC (LGC Standards GmbH, Wesel, Germany). All the cells in the experiments were confirmed as free of mycoplasma. The cells were cultured in RPMI-1640 (Gibco, USA) containing 10% fetal bovine serum (Sigma-Aldrich, USA) at 37 °C in a humidified atmosphere containing 5% CO_2_. The cells were treated with 5 μM 5-Aza-CdR (Sigma-Aldrich, USA) for 72 h. Then, the cells were collected for RNA and protein extraction.

### Western blotting

Western blotting was performed as described previously [30]. The cells were disrupted with ice-cold RIPA buffer with 2 mM PMSF and protease inhibitor cocktail (Merck, USA). The protein concentration was determined using a BCA Protein Assay Kit (Cwbio, Beijing, China). Total protein (10 μg) was separated by 10% TGX Stain-Free gels (Bio-Rad, USA) and transferred onto a nitrocellulose membrane (Millipore, USA). 5% skim milk was used to block the membranes for 1 h at room temperature. The membrane was cropped according to predicted position of target protein. Subsequently, membranes were incubated with primary antibodies at 4 °C overnight and then incubated with specific IRDye 800CW-conjugated antibodies (Odyssey, USA, 1:10000) after washing with PBST three times. Primary antibodies used were rabbit monoclonal α-Syn (ab138501, Abcam, USA, 1:1000); mouse monoclonal GAPDH (60004–1-Ig, Proteintech™, China, 1:20000). The bands were visualized using the Odyssey imaging system (Licor, USA). This system produces a signal number for each band identified on a western blot generated by the near-infrared fluorescent detection of secondary antibodies used and provides low autofluorescence, higher sensitivity and multiplex labeling [31]. The densitometric analyses of the blots were performed using Image J software. GAPDH was used as a loading control. The *t* test was used to estimate the significance of difference in protein expression levels between groups. * *p* < 0.05; ** *p* < 0.01.

### Statistical analysis

The Student’s *t* test was used to compare the two groups. *P* values less than 0.05 were considered statistically significant and marked as follows: * *p* < 0.05, ** *p* < 0.01, *** *p* < 0.001, **** *p* < 0.0001. The survival analysis was obtained using log-rank test, and the correlations of *SNCA* with immune infiltration and specific immune cell markers were evaluated using Spearman’s correlation.

## Results

### Downregulated expression of *SNCA* in BLCA

**Fig. 1:**
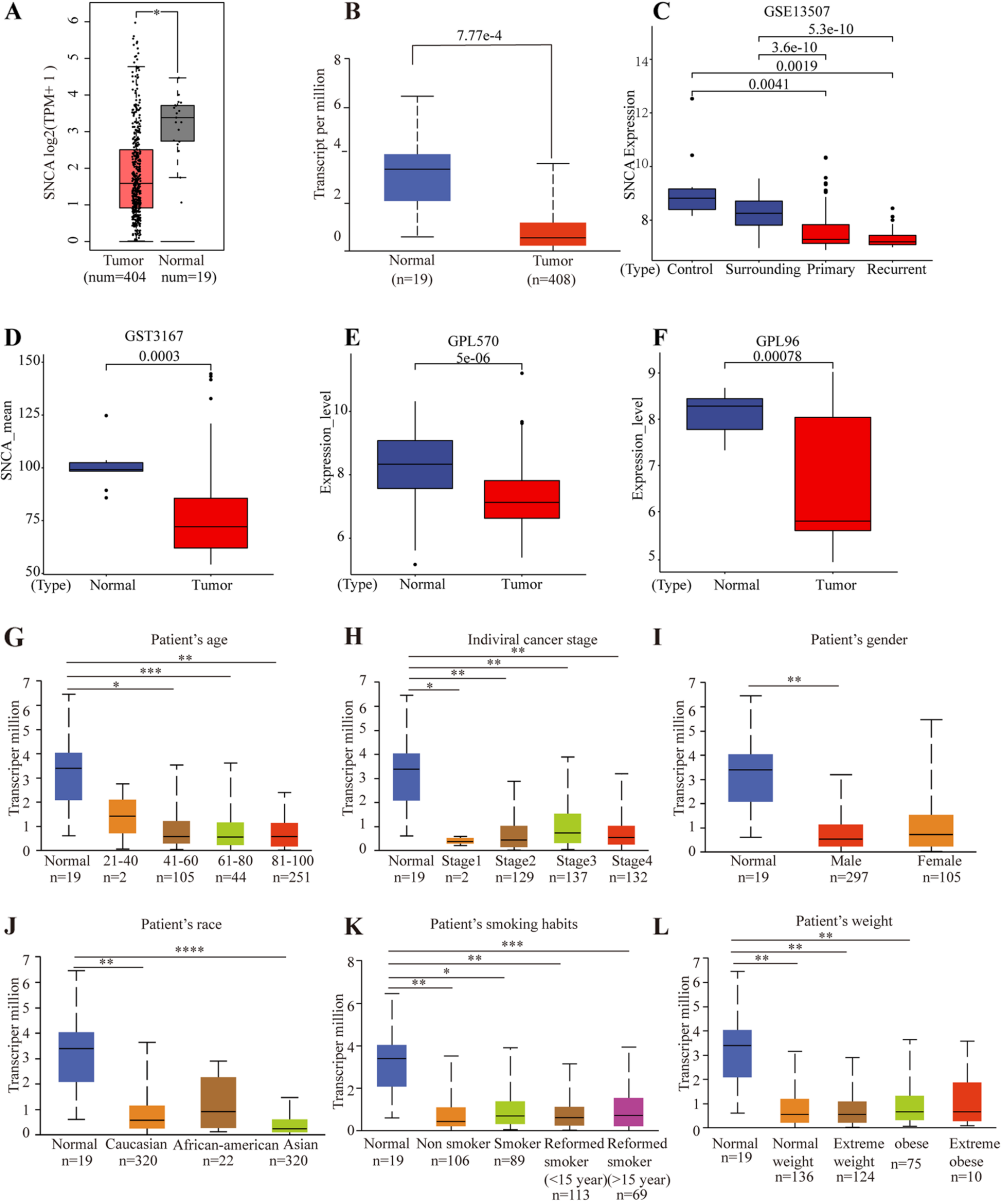
*SNCA* transcriptional level in BLCA is downregulated. **A** Box-whisker plots showing the expression levels of *SNCA* in tumor tissues and normal tissues, according to *t* test in TCGA by GEPIA2. **B** Box-whisker plots showing the expression levels of *SNCA* in tumor tissues and the normal tissues, according to *t* test in TCGA by UALCAN. **C-F** The expression levels of *SNCA* in tumor tissues and the normal tissues from GSE13507, GSE3167, GPL570 and GPL96. **G-L** Box-whisker plots showing the *SNCA* transcription in subgroups of patients with BLCA, stratified based on age (**G**), individual cancer stage (**H**), gender (**I**), race (**J**), smoking habits (**K**), and body weight (**L**). The *t* test was used to estimate the significance of difference in gene expression levels between groups. *, *p* < 0.05; **, *p* < 0.01; ***, *p* < 0.001; ****, *p* < 0.0001

We initially evaluated the *SNCA* transcription levels (TPM) of 404 BLCA tissue samples and 19 normal samples from TCGA and GTEx by setting the threshold log2|FC| = 1 and *P*-value = 1e-6 in GEPIA2 database, and found that the *SNCA* mRNA was significantly less expressed in BLCA tissues (Fig. 1A). The same significant result (*P*-value = 7.77e-4) was obtained from UALCAN database (Fig. 1B). Moreover, we further studied *SNCA* expression base on GSE13507 and GSE3167 from GEO database, these datasets showed that the mRNA expression of *SNCA* were obviously lower in BLCA patients compared with normal tissues (Fig. 1C, D). In GENT2 database, we downloaded *SNCA* expression in GPL570 and GPL96, and we got the same result as mentioned above, the significance *p*-value is 5E-6 and 7.8E-4, respectively (Fig. 1E, F).

Further subgroup analysis of multiple clinic-pathological features of TCGA-BLCA samples in UALCAN database consistently showed the downregulated transcription level of *SNCA* in BLCA patients compared with that in normal subjects based on age (Fig. 1G), stage (Fig. 1H), gender (Fig. 1I), race (Fig. 1J), smoking habits (Fig. 1K), and weight (Fig. 1L). However, there were no differences in transcription level of *SNCA* between the female patients and normal (Fig. 1I). As reported by Pravin that men have a 3–4 time higher incidence of BLCA than women [32]. It is indicated expression of *SNCA* may serve as a potential diagnostic indicator of BLCA.

### High expression of *SNCA* is an independent risk factor for OS

**Fig. 2:**
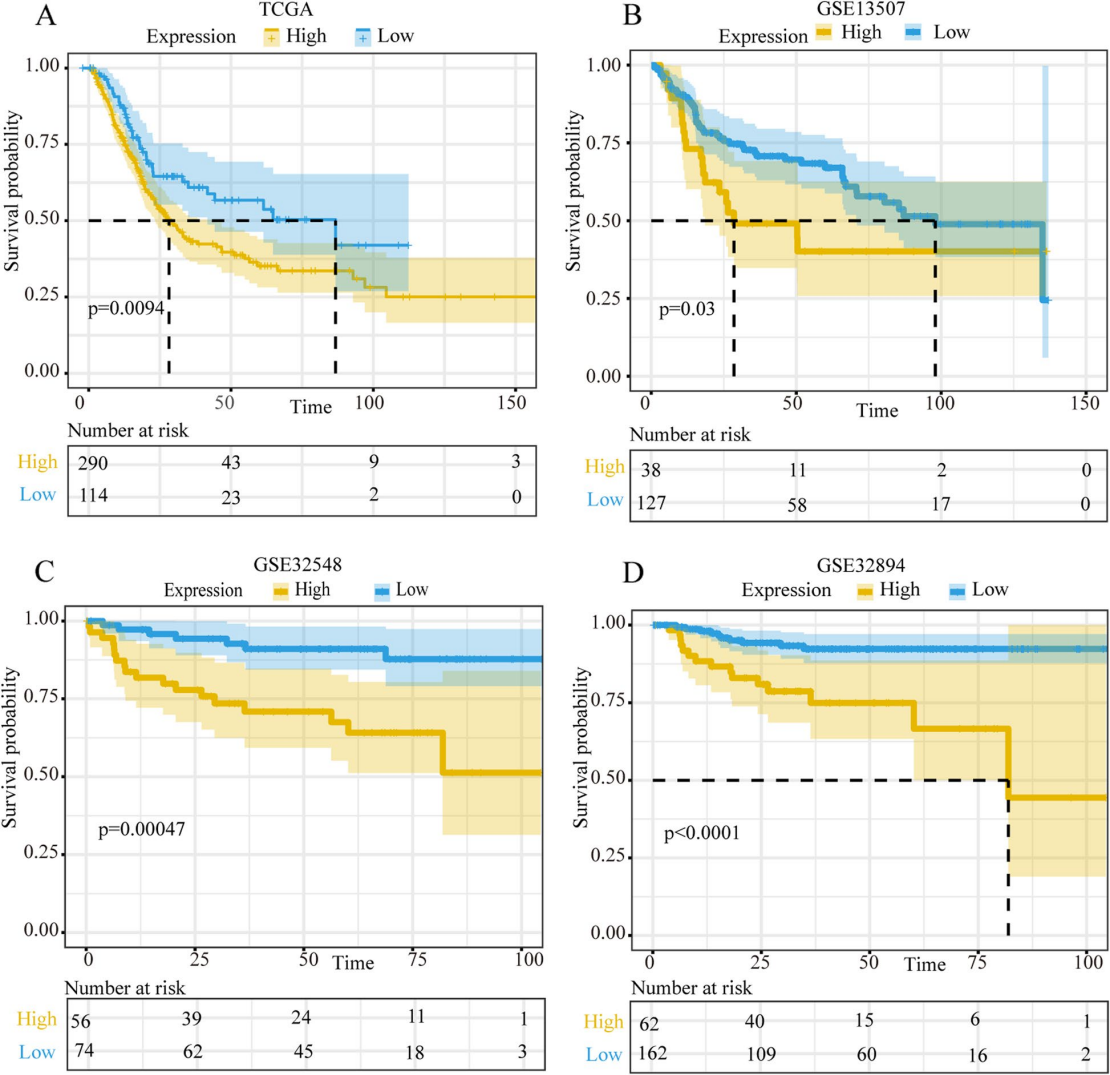
*SNCA* is associated with survival outcome. **A** Overall survival (OS) of *SNCA* in TCGA BLAC cohort. **B-D** OS of *SNCA* analysis from Kaplan–Meier survival curves on GSE13507 **(B**), GSE 32548 (**C**), and GSE 32894 (**D**). The numbers below the figures denote the number of patients at risk in each group. The survival analysis was obtained using log-rank test

For different expression levels of *SNCA* in BLCA, we further analyzed the relationship between *SNCA* expression and prognosis in individuals with BLCA. The patients were classified to groups according to the *SNCA* optimal grouping threshold, which was found by R package “survminer”. The results from TCGA based on 404 cases of BLCA patients showed that the low *SNCA* expression (FPKM) group had significantly longer OS (log-rank test, *p* < 0.05) compared with the high expression group in BLCA cohort (Fig. 2A). Similarly, in independent cohorts from GEO (GSE13507, GSE32548 and GES32894) (Fig. 2B, C, D), the low expression group had significantly more favorable OS than the high expression group. Hence, the high expression of *SNCA* was an independent risk factor for worse OS. It can be suggested that the high expression of *SNCA* can be used as a prognostic marker in BLCA, which is worth further clinical verification.

### *SNCA* co-expression networks in BLCA

**Fig. 3:**
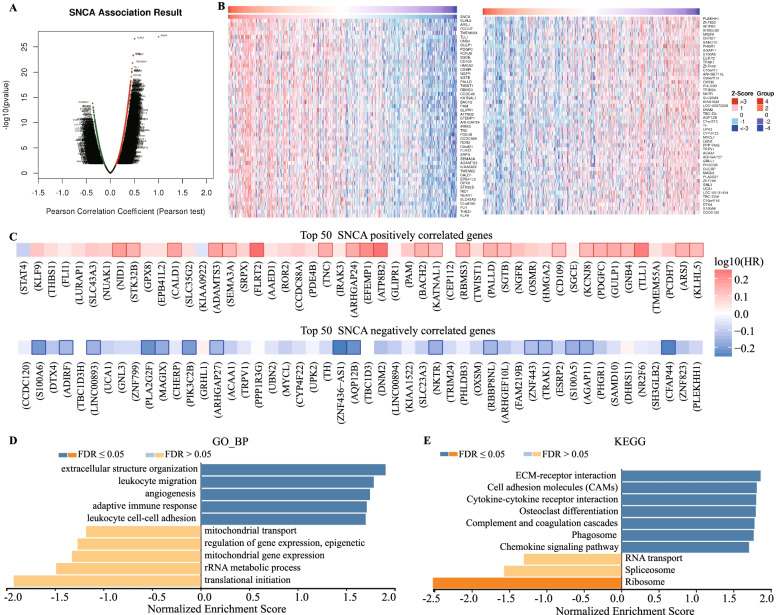
Co-expression genes of *SNCA* in BLCA analyzed by LinkedOmics database. **A** All genes highly correlated with *SNCA* as identified by Pearson correlation test in the BLCA cohort. **B** Heat maps showing the top 50 genes positively and negatively correlated with *SNCA* in BLCA. Red indicates positively correlated genes, and blue indicates negatively correlated genes. **C** Survival heat maps of the top 50 significant genes positively and negatively correlated with *SNCA* in BLCA. The survival heat maps show the hazard ratios in the logarithmic scale (log10) for different genes. The red and blue blocks denote higher and lower risks, respectively. The rectangles with frames indicate the significant unfavorable and favorable results in prognostic analyses (*p* < 0.05). **D – E** Significantly enriched GO annotations and KEGG pathways of *SNCA* in the BLCA cohort

To gain insights into the biological meaning of *SNCA* in BLCA, the function module of LinkedOmics was applied to examine the co-expression pattern of *SNCA*. As shown in Fig. 3A, 4335 genes (dark red dots) showed significant positive correlation with *SNCA*, while 2184 genes (dark green dots) had significant negative correlation (FDR < 0.01). The detailed description of the co-expressed genes is provided in Supplementary Table S2. The top fifty significant genes positively or negatively associated with *SNCA* are shown by heat map in Fig. 3B. *SNCA* expression showed a strong positive relationship with the expression levels of *KLHL5* (*r* = 0.501, *p* = 2.45E-27), *ARSJ* (*r* = 0.473, *p* = 3.95E-24) and *PCDH7* (*r* = 0.472, *p* = 4.96E-24), and strong negative associations with *PLEKHH1* (*r* = − 0.368, *p* = 1.46E-14), *ZNF*823 (*r* = − 0.358, *p* = 8.50E-24) and *CFAP44* (*r* = − 0.356, *p* = 1.22E-13). Remarkably, the top 50 significantly positive genes showed a high probability of becoming high-risk markers in BLCA, in which 23/50 genes were with high hazard ratio (HR, *p* < 0.05) and while 16/50 genes were with low HR in the top fifty negatively significant genes (Fig. 3C). The outcomes of the GO analysis carried out by GSEA showed that *SNCA* co-expressed genes were involved mainly in the extracellular structure organization, leukocyte migration, angiogenesis, adaptive immune response, and leukocyte cell–cell adhesion (Fig. 3D and Supplementary Table S3). The KEGG pathway analysis showed enrichment in ECM-receptor interaction, cell adhesion molecules, cytokine–cytokine receptor interaction, and complement and coagulation cascades (Fig. 3E and Supplementary Table S4). These results suggest an impact of *SNCA* on cell migration.

### Identification of crucial modules and hub genes in each module co-regulated with survival in BLCA by WGCNA analysis

**Fig. 4:**
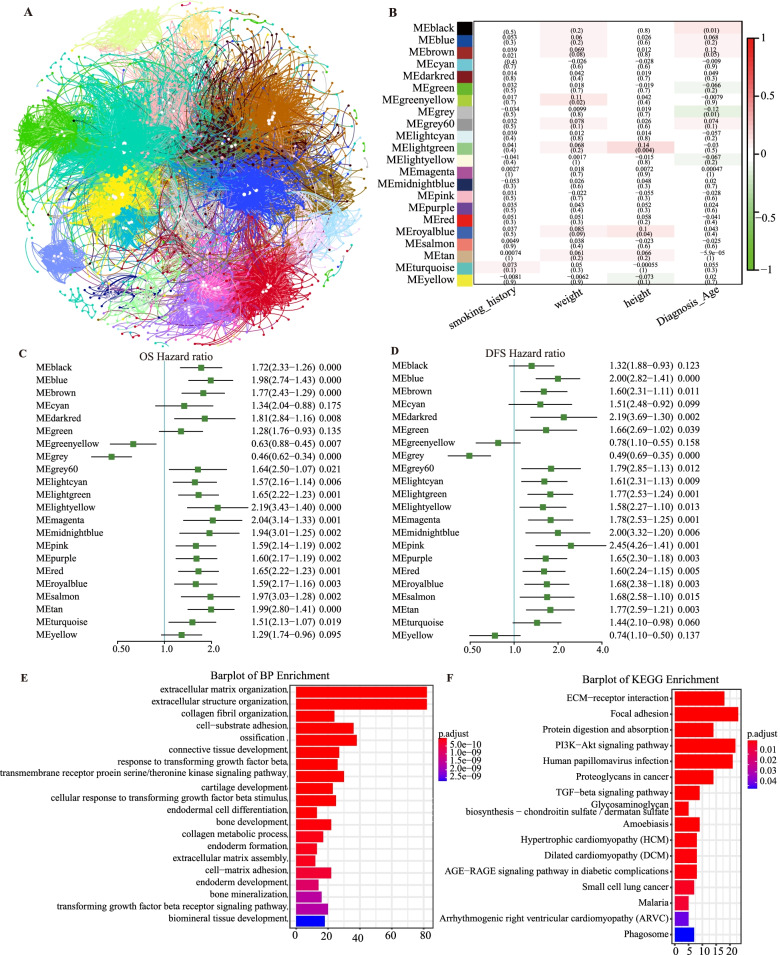
Identification of *SNCA* co-expression key modules by WGCNA in BLCA. **A** The unsupervised hierarchical cluster dendrogram was used to identify co-expression modules and assign colors to them. A total of 22 modules were identified and represented by different colors. The white circles indicate the top five hub genes. **B** Spearman correlation matrix heat map of the correlation module MEs and clinical traits (smoking habits, weight, height, and age at diagnosis). The darker the module color, the more significant the relationship. **C-D** Multivariate hazards models were used to evaluate the effects of the modules on OS and DFS. **E** GO analysis of genes involved in the brown module in terms of biological process, cellular component, and molecular function. **F** KEGG analysis of genes involved in the brown module

To further interpret the functions of *SNCA* in BLCA. We choose the highly correlated genes defined before to construct the weighted gene co-expression network using WGCNA. WGCNA is a systems biology method for an overview of the transcriptomic organization, and the relationships between sets of genes with external biological traits [26]. We constructed the gene network and identified modules using the one-step network construction function of WGCNA R package for the co-expressed genes screened by LinkedOmics as mentioned above (FDR < 0.01) (Supplementary Table S2). Twenty-two gene co-expression modules were constructed. To understand the functions of these interesting modules, top five hub genes in each module except MEgrey module (this module contains the genes which have not been clustered in any module) were selected, which were highlighted in white dot (Fig. 4A). These hub genes were defined as genes with high connectivity and high module membership (MM > 0.8) in these identified modules, and totally 104 hub genes were obtained. The detailed information for all identified hub genes was provided in Supplementary Table S5. Then, the relationship between these hub genes’ expression and prognosis in individuals with BLCA were analyzed, and we found that more than 70% (76/104) genes are significantly correlated with prognosis in OS, and all the results were provided in Supplementary Table S6. Moreover, the relationship between modules and clinical traits, including the patients’ smoking habits, weight, height, and age at diagnosis, were evaluated. Interestingly, the results showed that most of these traits did not highly significantly correlate with these modules. Even the highest correlation score that between the light-green module and height (*r* = 0.14, *p* = 0.004) was moderate (Fig. 4B). Other clinical characteristics (stage, race, grade, gender, and diagnosis subtype) correlating with the modules are shown in Supplementary Fig. S1.

Given that we had previously found that high *SNCA* expression indicated worse survival in individuals with BLCA (Fig. 2), the multivariate hazards models were used to evaluate the correlations between the 22 modules and OS or DFS. The forest maps showed that most modules significantly affected OS and DFS (HR > 1, *p* < 0.05) (Fig. 4C and D). Specifically, the modules of blue, brown, dark, grey, grey60, light cyan, light green, light yellow, magenta, pink, purple, red, royal blue, salmon, and tan significantly correlated with both OS and DFS (Fig. 4C and D). It is indicated that *SNCA* was a factor of poor survival. The module brown was selected to perform the functional analyses based on the GO and KEGG databases. According to the results of these analyses, the genes were mainly enriched in extracellular matrix organization, extracellular structure organization, cell–substrate adhesion, and ossification (Fig. 4E). We then performed the KEGG pathway analysis of the genes in the brown module and identified the module-regulated pathways. The results of the analysis showed that the brown-module-regulated pathways included cell adhesion, PI3K-Akt signaling pathway, and TGF-beta signaling pathway, which all play an important role in cancer processing (Fig. 4F).

### The transcription level of *SNCA* is associated with tumor immune infiltration

**Fig. 5:**
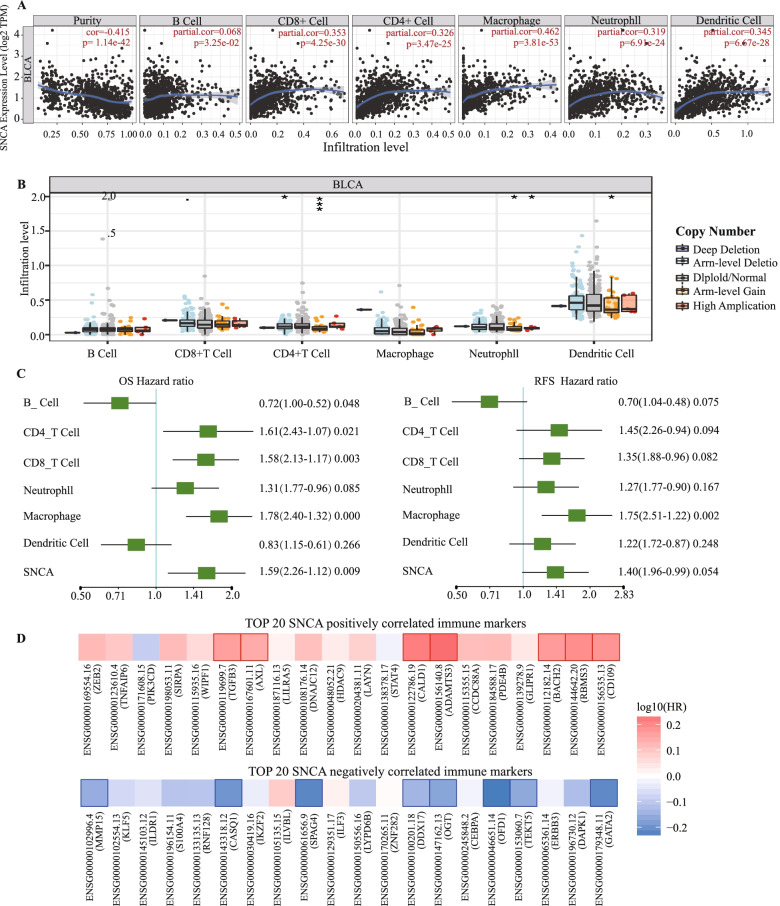
Correlations of *SNCA* expression with immune infiltration level in BLCA. **A** *SNCA* expression negatively correlated with the tumor purity and showed significant positive correlations with the infiltrating levels of CD8+ T cells, CD4+ T cells, macrophages, neutrophils, and dendritic cells in BLCA. **B** *SNCA* CNV affected the infiltrating levels of CD4+ T cells, neutrophils, and dendritic cells. **C** Multivariate hazards models were used to evaluate the effects of *SNCA* expression on OS and RFS in the presence of multiple immune cells infiltrates. **D** Survival heat maps of the top 20 immune markers in BLCA that were positively and negatively correlated with *SNCA*. The red and blue blocks denote higher and lower risks, respectively. The rectangles with frames indicate the significant unfavorable and favorable results in prognostic analyses (*p* < 0.05)

Tumor-infiltrating immune cells are a part of the complex microenvironment and are associated with the biological behavior and patient survival in BLCA [33]. We further investigated whether *SNCA* expression correlated with the six main immune infiltration cells (B cells, CD4+ T cells, CD8+ T cells, neutrophils, macrophages, and dendritic cells) in BLCA from TIMER database. The results showed that the expression level of *SNCA* correlated negatively with the tumor purity (*r* = − 0.415, *p* = 1.14E-42) and positively with CD8+ cell (*r* = 0.353, *p* = 4.25E-30), CD4+ cells (*r* = 0.326, *p* = 3.475E-25), macrophages (*r* = 0.462, *p* = 3.81E-53), neutrophils (*r* = 0.319, *p* = 6.91E-24), and dendritic cells (*r* = 0.345, *p* = 6.67E-28) (Fig. 5A). Particularly, Copy number variations (CNV) of *SNCA* were significantly associated with the infiltrating levels of CD4+ cells, neutrophils, and dendritic cells (Fig. 5B). Given that the expression of *SNCA* was related to the immune infiltration in BLCA, and that *SNCA* expression was also an independent risk factor for OS, we speculated that *SNCA* expression affected the prognosis of patients with BLCA partly due to the immune infiltration. Multivariate hazards models were used to evaluate the effects of *SNCA* expression in the presence of six types of immune cells on the OS and RFS. As shown in Fig. 5C, *SNCA* had 1.59 times higher risks on OS (*p* = 0.009) and 1.40 times higher risks on RFS (*p* = 0.054). In particular, T cell infiltration seemed to be a critical factor for *SNCA* to increase the risk of poor OS (HR > 1, *p* < 0.05) (Figs. 5C). Survival map analysis showed the high risk of *SNCA* positively correlated with the marker genes and the low risk of *SNCA* negatively correlated with the marker genes (Fig. 5D). Generally, the top five markers positively correlated with *SNCA* were *ZEB2*, *TNFAIP6*, *PIK3CD*, *SIRPA*, and *WIPF1*, whereas *GATA2*, *DAPKI*, *ERBB3*, *TEKT5*, and *OFD1* were the top five negatively correlated markers genes (Fig. 5D). Collectively, these results indicate that *SNCA* expression may affect prognosis of patients with BLCA, at least partially due to the immune infiltration.

### *SNCA* expression is associated with immune signatures

We further explored the relationships between *SNCA* and the markers of different immune cell types. We analyzed the correlations between *SNCA* expression and various immune signatures, including immune marker genes of 28 tumor-infiltrating lymphocytes (TILs), immune inhibitory or stimulatory, cytokine-related, cancer-testis antigen, and MHC genes. The analysis after the correlation adjustment by tumor purity, we showed that the expression level of *SNCA* in BLCA significantly correlated with 40.27% (611/1578) of the immune marker genes (Supplementary Table S7). Among the significantly correlated immune markers, 77.41% (473/611) correlated positively and 22.59% (138 / 611) correlated negatively. The top five marker genes positively correlated with *SNCA* were *CCDC88A* (*r* = 0.403, *p* = 7.90E - 16), *ELVOL4* (*r* = 0.384, *p* = 2.20E - 14), *DNAJC12* (*r* = 0.382, *p* = 3.03E - 14), *RBMS3* (*r* = 0.380, *p* = 4.09E - 14), and *HDAC9* (*r* = 0.376, *p* = 8.15E - 14). The top five markers negatively correlated with *SNCA* were *S100A4* (*r* = − 0.241, *p* = 2.86E - 06), *TEKT5* (*r* = − 0.212, *p* = 3.97E - 05), *CEBPA* (*r* = − 0.210, *p* = 4.89E - 05), *GATA2* (*r* = − 0.209 *p* = 5.26E- 05), and *LYPD6B* (*r* = − 0.204, *p* = 7.71E - 05) (Supplementary Table S7).

As for immunoinhibitory genes, we found that *IL10* and *CSF1R* had a positive correlation with *SNCA* expression (Supplementary Table S7). The *IL10* is a key anti-inflammatory mediator ensuring protection of a host from over-exuberant responses to pathogens and microbiota, while playing important roles in immunotherapy for cancer [34]. *CSF1R* is reported correlation with poor survival in various tumor types. Targeting *CSF1R* signaling in tumor-promoting TAM represents considered an attractive strategy to eliminate or repolarize macrophages cells [35]. Moreover, the top five immunostimulatory genes positively correlated with *SNCA* were *TMEM173*, *ENTPD1*, *IL6*, *IL6R*, and *MICB*. There are no immunoinhibitory and immunostimulatory genes that negatively correlated with *SNCA* expression (Supplementary Table S7).

In the previous section, we showed that T cell infiltration may be one of the key reasons for the prognostic value of *SNCA* (Fig. 5B). Therefore, we examined the correlation between *SNCA* and T cell marker genes. Table 2, which were an extract from Supplementary Table S7, showed the purity-corrected partial Spearman’s correlation coefficients between *SNCA* and T cell markers. In total, 33/51 of the T cell marker genes were significantly associated with *SNCA* expression; the numbers of positive and negative correlations were 32/33 (96.96%) and 1/33 (3.03%), respectively. In activated CD8+ T cells, *SNCA* highly correlated with protein *MPZL1*. Indeed, *MPZL1* has been reported to promote tumor cell migration via the Src signaling pathway [36, 37]. In activated CD4+ T cells, *SNCA* significantly correlated with *ETS1*, which has been predicted to play an inhibitory role in cell migration in BLCA [38, 39].

**Table 2:** Correlation analysis between *SNCA* and markers of activated T cells

Actived CD8 T cell	None		Purity		Actived CD4 T cell	None		Purity	
	cor	p	cor	p		cor	p	cor	p
ADRM1	0.007007181	0.893285143	0.011691546	0.823125096	AIM2	0.184848948	0.000357535	0.110447477	0.034172889
AHSA1	0.032991678	0.527536367	0.049624408	0.342464207	BIRC3	0.276706999	6.55E-08	0.157768453	0.002403299
C1GALT1C1	0.072903957	0.162248803	0.082411178	0.114513305	BRIP1	0.116107671	0.025725106	0.144358809	0.005530239
CCT6B	−0.04923596	0.345601564	− 0.024851383	0.634656036	CCL20	0.245419021	1.83E-06	0.18265844	0.000428711
CD37	0.313734339	7.16E-10	0.160668404	0.001989611	CCL4	0.260407905	3.91E-07	0.117518488	0.024162004
CD3D	0.181560811	0.000456589	0.02477063	0.635757247	CCL5	0.173718161	0.000804592	0.007936149	0.8794029
CD3E	0.261800098	3.38E-07	0.105558417	0.04299798	CCNB1	0.117676015	0.023779521	0.12614563	0.015463408
CD3G	0.237820179	3.85E-06	0.10315968	0.047986046	CCR7	−0.036747062	0.481600452	−0.094694125	0.069608871
CD69	0.289939997	1.40E-08	0.17500006	0.000747011	DUSP2	0.007569049	0.884785523	0.048693839	0.351602967
CD8A	0.249516366	1.21E-06	0.122661758	0.018575071	ESCO2	0.116216223	0.025586159	0.137595042	0.008214262
CETN3	0.096863333	0.063063465	0.170969376	0.000991666	ETS1	0.321388314	2.60E-10	0.246498672	1.70E-06
CSE1L	0.099792461	0.055463188	0.103048443	0.048228643	EXO1	0.179712783	0.000522907	0.193044649	0.00019474
GEMIN6	0.050822929	0.330258387	0.063946265	0.221034641	EXOC6	0.09118768	0.080228582	0.137066124	0.008466521
GNLY	0.22254285	1.60E-05	0.090152375	0.084157409	IARS	0.150831049	0.003682261	0.121615747	0.019610062
GPT2	−0.161011684	0.001917589	−0.111822512	0.03198804	ITK	0.283888625	2.87E-08	0.140204208	0.007065302
GZMA	0.237857212	3.84E-06	0.097395644	0.061977642	KIF11	0.106512789	0.040862497	0.135977526	0.009007528
GZMH	0.178901654	0.000554748	0.032912338	0.529093309	KNTC1	0.062290422	0.23261112	0.134880144	0.009583803
GZMK	0.263285516	2.88E-07	0.115159561	0.027175914	NUF2	0.071277459	0.171848539	0.113930413	0.028870616
IL2RB	0.273384628	9.52E-08	0.136656971	0.008666369	PRC1	0.081499059	0.118091424	0.111232326	0.032910755
LCK	0.243508727	2.21E-06	0.117150056	0.024612812	PSAT1	0.072592038	0.164057841	0.068861898	0.187479816
MPZL1	0.301591522	3.38E-09	0.24175371	2.71E-06	RGS1	0.345455988	8.81E-12	0.221428298	1.81E-05
NKG7	0.220843391	1.86E-05	0.06600439	0.206497901	RTKN2	0.08175509	0.116936899	0.124763933	0.016638133
PIK3IP1	0.00561427	0.914406956	0.013977917	0.789281481	SAMSN1	0.355518658	1.96E-12	0.228851868	9.24E-06
PTRH2	−0.025229406	0.629043428	−4.85E-05	0.999259876	SELL	0.155131466	0.002808311	0.079284636	0.128974215
TIMM13	−0.10305631	0.047906098	−0.064104898	0.219888868	TRAT1	0.246269682	1.68E-06	0.106436583	0.041284495
ZAP70	0.248801177	1.30E-06	0.109145152	0.03635807					

Cor, R value of Spearman’s correlation; None, correlation without adjustment. Purity, correlation adjusted by purity. * *p* < 0.05

### The expression of *SNCA* and key genes detected in patients with BLCA

**Fig. 6:**
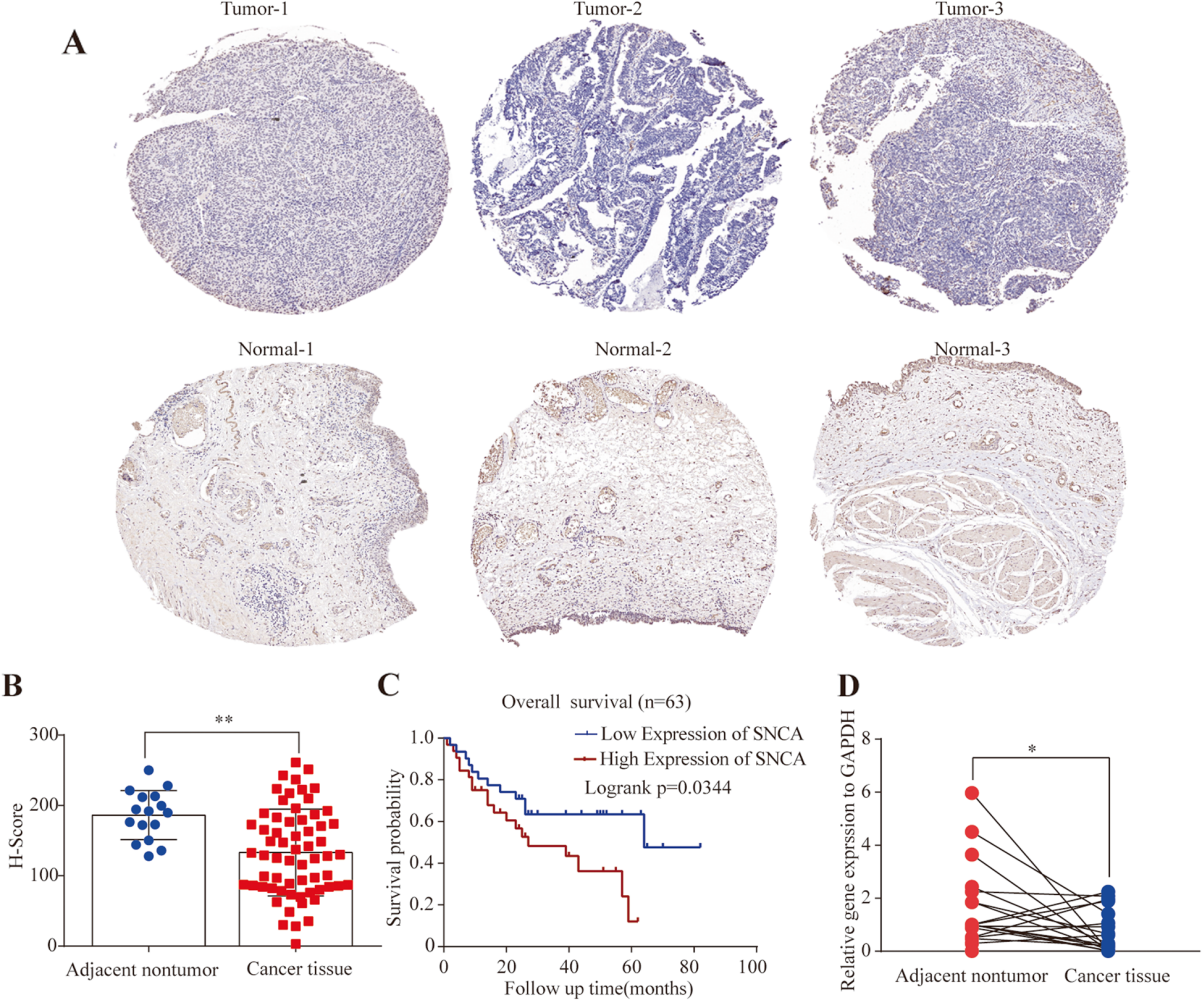
Expression of *SNCA* detected in BLCA patients. **A** Representative images for the protein levels of α-Syn investigated by IHC. **B** The histochemistry score (H-score) of *SNCA* expression analysis by Image-Pro Plus 6.0 showed that *SNCA* was significantly downregulated in the BLCA tissue array (*n* = 63). *P* -value represents unpaired Student’s *t* test, **p < 0.01. **C** Kaplan–Meier analysis of the correlation between *SNCA* expression and the OS of patients with BLCA in the tissue array (*n* = 63). **D** *SNCA* mRNA expression was significantly downregulated in BCa compared with para-cancer tissues in our cohort, as determined by qRT-PCR (*n* = 20). *SNCA* expression was normalized to *GAPDH*. *P*-value represents paired Student’s *t* test, *p < 0.05

To verify *SNCA* expression in BLCA, we performed IHC staining on the tissue microarray containing samples from 63 cases of BLCA and paired adjacent nontumor tissues purchased from Outdo Biotech (Supplementary Table S8). The results showed that the protein level of α-Syn was significantly downregulated in primary BLCA tissues compared with adjacent non-tumor samples (*p* < 0.01) (Fig. 6A and B). The prognostic value of *SNCA* was further investigated in these patients. As shown in Fig. 6C, the BLCA patients with high *SNCA* expression exhibited significantly shorter OS than those with low *SNCA* expression (Figs. 6C). We then performed qRT-PCR analysis on 20 pairs of BLCA and para-cancer tissue samples of our cohort at the transcription level. *SNCA* expression was significantly lower in BLCA tissues than in para-cancer tissues (*p* < 0.05) (Fig. 6D). These data confirmed the downregulated expression of *SNCA* in BLCA, which was consistent with the results from TCGA and GEO cohorts.

**Fig. 7:**
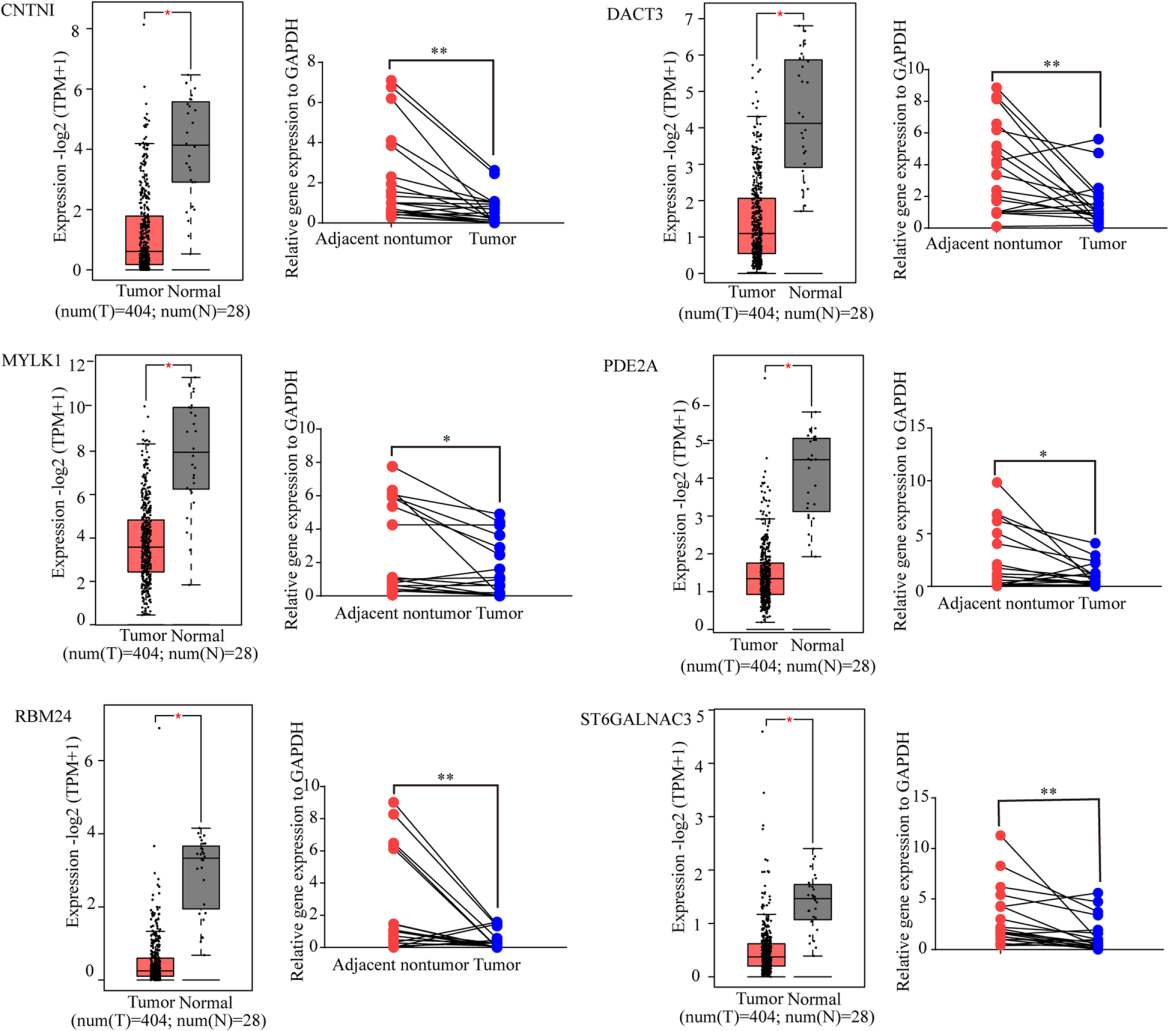
Key gene expressions in BLCA. Box plot showed the relative expression of *SNCA* in BLCA patients in the GEPIA2. The right image showed the relative expression of *SNCA* in 20 pairs of BLCA tissues analyzed by qRT-PCR. *SNCA* expression was normalized to *GAPDH*. *P*-value represents paired Student’s *t* test, *p < 0.05, ** *p* < 0.01

Total of 20 genes among the hub genes were significantly correlated with OS and DFS (Supplementary Table S6), of which the six key genes screened by univariate Cox analysis, which were significant risk factors for OS in BLCA, were *CNTN1*, *DACT3*, *MYLK1*, *PDE2A*, *RBM24*, and *ST6GALNAC3* (*p* < 0.01) (Table 3 and Supplementary Fig. S2). These six genes expression of 404 BCLA tissues and 28 normal bladder specimens from TCGA were analyzed with GEPIA2. We found the all the six genes were significantly downregulated in BLCA (Fig. 7). The results were confirmed by qRT-PCR for the 20 pairs of BLCA and para-cancer tissue samples of our cohort (Fig. 7).

**Table 3:** Univariate Cox analysis for key genes

Characteristics	Total(N)	Univariate analysis	
		Hazard ratio (95% CI)	***P*** value
ADGRL4 (Low vs. High)	407	0.709 (0.527–0.955)	**0.024**
ANGPTL1 (Low vs. High)	407	0.694 (0.515–0.935)	**0.016**
CDH5 (Low vs. High)	407	0.789 (0.587–1.060)	0.116
CNN1 (Low vs. High)	407	0.727 (0.539–0.981)	**0.037**
CNTN1 (Low vs. High)	407	0.672 (0.497–0.907)	**0.009**
DACT3 (Low vs. High)	407	0.658 (0.487–0.890)	**0.007**
DIPK2B (Low vs. High)	407	0.722 (0.537–0.970)	**0.031**
BMX (Low vs. High)	407	0.712 (0.530–0.957)	**0.024**
F13A1 (Low vs. High)	407	0.691 (0.513–0.931)	**0.015**
KCNH2 (Low vs. High)	407	0.902 (0.672–1.211)	0.493
KCNMB1 (Low vs. High)	407	0.790 (0.587–1.064)	0.121
LMOD1 (Low vs. High)	407	0.690 (0.512–0.930)	**0.015**
MMP9 (Low vs. High)	407	0.707 (0.525–0.952)	**0.022**
MYLK (Low vs. High)	407	0.577 (0.426–0.782)	**< 0.001**
PDE2A (Low vs. High)	407	0.632 (0.469–0.850)	**0.002**
PDE5A (Low vs. High)	407	0.774 (0.574–1.044)	0.093
RBM24 (Low vs. High)	407	0.668 (0.496–0.900)	**0.008**
ST6GALNAC3 (Low vs. High)	407	0.642 (0.477–0.865)	**0.004**
TAP2 (Low vs. High)	407	0.920 (0.685–1.236)	0.580
WNT5B (Low vs. High)	407	0.704 (0.523–0.948)	**0.021**

### The lower expression of *SNCA* in BLCA is related to its promoter hypermethylation

**Fig. 8:**
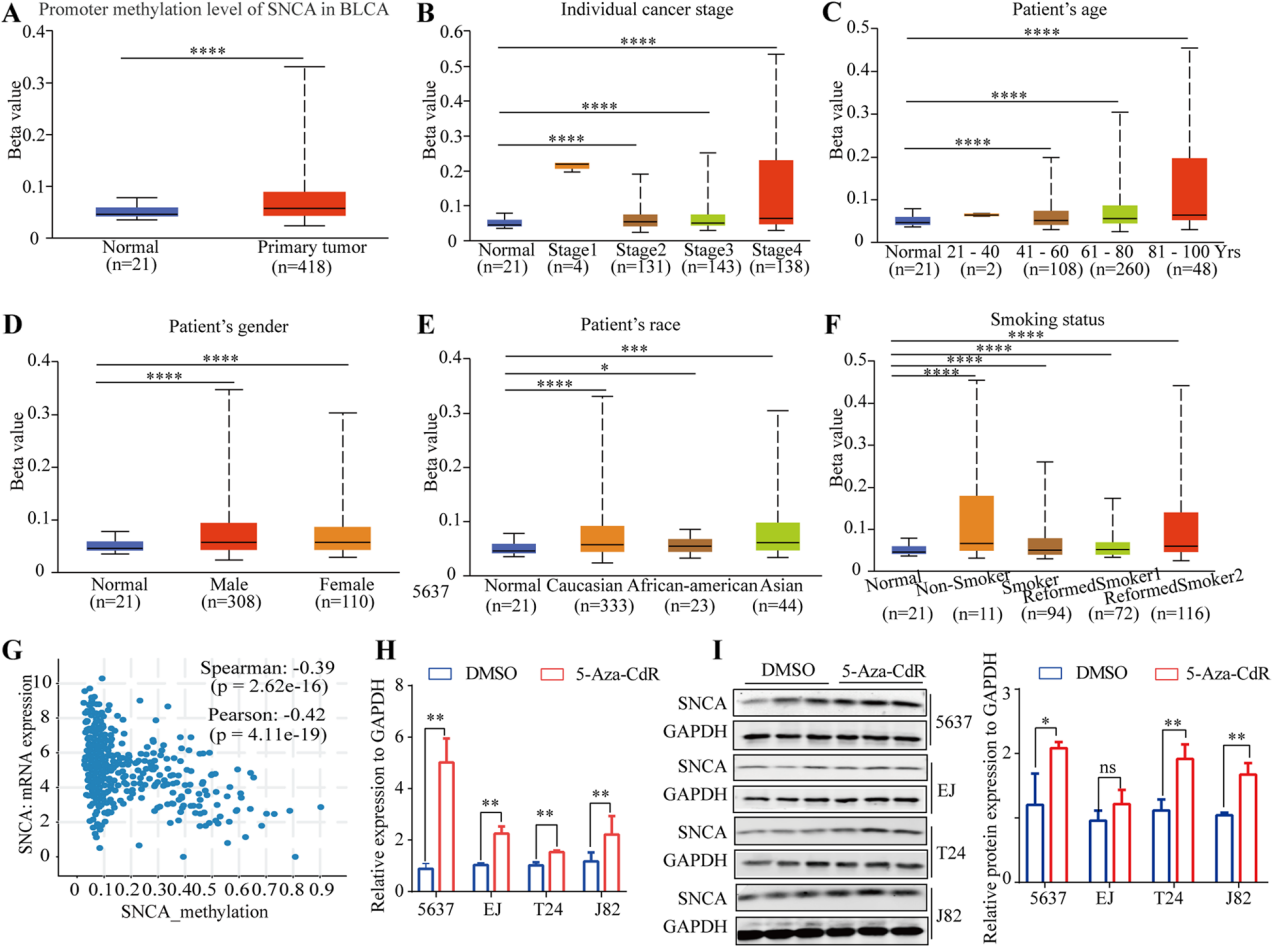
The promoter methylation level of *SNCA* is upregulated in BLCA and negatively correlates with the expression level of *SNCA*. **A** The *SNCA* promoter methylation level of BLCA patients from TCGA analyzed by UALCAN. The expression level of *SNCA* methylation in BLCA patients downloaded from UALCAN database was analyzed according to (**B**) cancer stage, (**C**) patient’s age, (**D**) gender, (**E**) race, and (**F**) smoking status. **G** The correlation analysis between the expression level of *SNCA* and its methylation level in BLCA patients. **H** qRT-PCR analysis of the expression of *SNCA* in BLCA cell lines treated with 5-Aza-CdR in a final concentration of 5 μM for 72 h. **I** Western blotting analysis of the expression of *SNCA* in BLCA cell lines treated with 5-Aza-CdR in a final concentration of 5 μM for 72 h. *GAPDH* was used as a loading control. Quantitative analysis results are shown on the right

Aberrant DNA methylation is the most well studied epigenetic change, which culminates in altered gene expression, thereby playing critical roles in the initiation and progression of carcinogenesis [40]. We speculated that the downregulation of *SNCA* in BLCA may be related to DNA methylation. The promoter methylation level of *SNCA* in BLCA was investigated. As expected, the promoter methylation levels of *SNCA* were significantly higher in primary tumors than in normal tissues from UALCAN database (Fig. 8A). Further study based on subgroup analysis of multiple clinic-pathological features of TCGA-BLCA samples in UALCAN database consistently showed the higher promoter methylation levels of *SNCA* in BLCA patients compared with normal subjects based on cancer stage (Fig. 8B), age (Fig. 8C), gender (Fig. 8D), race (Fig. 8E), and smoking habits (Fig. 8F). As shown in Fig. 8G, the transcription level of *SNCA* negatively correlated with the level of promoter methylation analyzed from TCGA (Spearman: *r* = − 0.39, *p* = 2.62E - 16). To investigate whether the promoter methylation may serve as a regulator of the transcription of *SNCA* in BLCA, we treated the BLCA cell lines with 5-Aza-CdR, a demethylating agent approved by the U.S. Food and Drug Administration, at a final concentration of 5 μM for 72 h. The results showed significantly increased mRNA (Fig. 8H) and protein expression levels of *SNCA* in 5-Aza-CdR-treated group (Fig. 8I), indicating that *SNCA* is suppressed and downregulated due to its promoter hypermethylation.

## Discussion

Epidemiological studies have indicated a decreased risk of most cancer types in patients with PD. However, some tumors are more common in PD patients [41–43]. α-Syn has been reported to be abnormally expressed in a variety of tumors [5, 7–9], but it has never been studied in BLCA. In this study, we analyzed the clinical and prognostic role of *SNCA* as well as *SNCA* methylation in BLCA based on TCGA database. We found that the low expression of *SNCA* mRNA in BLCA tissues was significantly associated with a favorable prognosis. A negative association was found between *SNCA* mRNA expression and *SNCA* methylation. There is increasing evidence that abnormal DNA methylation of *SNCA* plays an essential role in a variety of tumors, such as cholangiocarcinoma [4], colonic adenocarcinoma [8], colorectal cancer [9], and non-Hodgkin lymphoma [14]. In our analysis, we first treated the BLCA cell lines with the DNA methylation inhibitor, 5-Aza-CdR. Such a treatment resulted in the significant increase in mRNA and protein expression levels of *SNCA*, indicating that *SNCA* may be suppressed and downregulated due to its hypermethylation. This negative correlation may well explain the low expression of *SNCA* in BLCA tissues. Thus, *SNCA* might be an independent biomarker for BLCA prognosis.

Cancer is one of the diseases resulting from incorrect signaling in cellular systems related to cell survival and death. α-Syn is mainly located in presynaptic nerve terminals and it plays an important regulatory role, including synapse maintenance, mitochondrial homeostasis, proteasome function, dopamine metabolism, and molecular chaperone activity [3]. At present, little is known as to how *SNCA* affects the prognosis of BLCA and what biological function *SNCA* has in BLCA. Both PD and cancer have been reported to share some common biological pathways in which *SNCA* is involved, such as mitochondrial dysfunction, inflammation, oxidative stress, DNA damage, and cell cycle activation anomalies [44]. LinkedOmics database was used to study the potential biological functions of *SNCA* in BLCA. The enrichment analysis of the genes co-expressed with *SNCA* showed that *SNCA* was significantly associated with cell adhesion, indicating an impact of *SNCA* on cell migration. A growing body of evidence indicates that *SNCA* interacts with actin and actin-binding proteins, whose rapid assembly and disassembly enable cells to migrate [45]. Both in vitro and in vivo model have shown that *SNCA* affects microtubules, which play many roles in all eukaryotic cell types from fungi to mammals [46]. *SNCA* overexpression correlates with disruption of the microtubule network, impairment of microtubule-dependent trafficking, and neurite degeneration [47]. It has been suggested that *SNCA* affects cell migration partly due to the interaction with actin; however, further mechanisms need to be confirmed by biological experiments.

WGCNA, a system biology method, can identify highly synergistic gene sets and can analyze the genes most relevant to disease based on the interlinkage of gene sets and the association between gene sets and phenotypes. In our study, WGCNA was used to cluster genes with similar expression patterns to obtain the modules most relevant to the clinical phenotype of BLCA patients. The results showed that most of the clinical characteristics (smoking habit, weight, height, ages at diagnosis, stage, race, grade, gender, and diagnosis subtype) did not significantly correlate with these modules. However, most modules significantly affected OS and DFS. These results together suggest that *SNCA* may be used as a prognostic marker in BLCA.

Tumor-infiltrating immune cells have emerged as a key regulator of tumor growth and progression. They are a part of the complex tumor microenvironment and are associated with the biological behavior and patient survival in BLCA [33]. In the present study, we found that *SNCA* expression positively correlated with the presence of CD8+ T cells (*r* = 0.353) and CD4+ T cells (*r* = 0.326) in the BLCA tissues. We further analyzed the cell markers of T cells, and obtained consistent results. Previous studies have reported that T lymphocyte infiltrates are present in parkinsonian brains and 1-methyl-4-phenyl-1, 2, 3, 6-tetrahydropyridine mouse model of PD [48, 49]. *SNCA* expression was correlated with infiltrating may take part in PD. It is indicated that the tight relationship between *SNCA* and T cell marker genes is the potential epicenter of the immune response and one of the critical factors affecting the prognosis.

It is worth noting that this analysis inevitably had limitations. First, the results originate from retrospective data, and more prospective data will be needed to prove the clinical utility of our findings. Second, although we preliminarily explored the biological roles of *SNCA* in BLCA through WGCNA analysis, the detailed mechanism linking *SNCA* expression and BLCA requires further biomedical experiments. Nevertheless, the current results are encouraging and noteworthy in the field of identifying promising prognostic biomarkers for BLCA.

## Conclusion

*SNCA* is downregulated in BLCA and is negatively regulated by DNA methylation in BLCA. Low *SNCA* expression predicts favorable prognosis in BLCA patients. Moreover, *SNCA* expression potentially contributes to the regulation of T cells, B cells, macrophages, neutrophils, and dendritic cells. Hence, *SNCA* probably plays an important role in the infiltration of immune cells, and could act as a promising prognostic biomarker in BLCA patients.

## Supplementary Information

**Additional file 1.**

**Additional file 2.**

**Additional file 3.**

**Additional file 4.**

**Additional file 5.**

**Additional file 6.**

**Additional file 7.**

**Additional file 8.**

**Additional file 9.**

**Additional file 10.**

**Additional file 11.**

## Data Availability

The datasets analyzed during the current study are available in the Linkedomics, TCGA database (https://tcga-data.nci.nih.gov/tcga/), TIMER database (https://cistrome.shinyapps.io/timer/), UALCAN database (http://ualcan.path.uab.edu/index.html), KEGG database (www.kegg.jp/kegg/kegg1.html), Kaplan–Meier survival curves (http://cran.r-project.org/web/packages/survival/index.html). c-BioPortal database (http://cbioportal.org), All data generated or analyzed during this study are included in this published article (and its supplementary information files). All the data were available from the corresponding authors for reasonable request.

## Abbreviations

PD: Parkinson’s disease
BLCA: Bladder cancer
OS: Overall survival
DFS: Disease-free survival
RFS: Recurrence-free survival
TCGA: The Cancer Genome Atlas
GEPIA: Gene Expression Profiling Interactive Analysis
GENT2: Gene Expression database of Nomal and Tumor tissues 2
GO: Gene Ontology
KEGG: Kyoto Encyclopedia of Gene and Genomes
GO_BP: GO biological process
WGCNA: Weighted Co-expression Network
MHC: Major histocompatibility complex
TILs: Tumor-infiltrating lymphocytes
qRT-PCR: Quantitative real-time polymerase chain reaction
IHC: Immunohistochemistry
CNV: Copy number variations
