# Supplementary material for: Prognostic significance of SNCA and its methylation in bladder cancer
Source: BMC Cancer. 2022 Mar 26;22:330. doi: 10.1186/s12885-022-09411-9 (PMC8961938; doi:10.1186/s12885-022-09411-9)
Supplement: Supplementary file 1 — Additional file 1. [file 12885_2022_9411_MOESM1_ESM.docx]

| **Gene name** | **primers** |
| --- | --- |
| CNTNI | Forward 5’- TGGCTGCTAAAGGTGGAAGG |
|  | Reverse 5’- TTGACAAGCCACTCTGTCCC |
| DACT3 | Forward 5’- TCTGCGAGTTACATTGGCTCC |
|  | Reverse 5’- CATCTTCATCCTCATCGGCGT |
| MYLK1 | Forward 5’- GCCCGCTCAATGCAGAAAAA |
|  | Reverse 5’- CTCAGCAACAGCCTCAAGGA |
| PDE2A | Forward 5’- GCCAGCAGGTCTTCCTCAAG |
|  | Reverse 5’- TCGATGACAGAGCCCAGACT |
| RBM24 | Forward 5’- GTACTTCGAGGTCTTCGGCG |
|  | Reverse 5’- AGGTTCACGTTGGCCTTTCT |
| ST6GALNAC3 | Forward 5’- ATGGCCTGCATCCTGAAGAG |
|  | Reverse 5’- GCACAACCAGCAGGAAAAGG |
| GAPDH | Forward 5’- GGAGCGAGATCCCTCCAAAAT |
|  | Reverse 5’- GGCTGTTGTCATACTTCTCATGG |
| SNCA | Forward 5’- AAGAGGGTGTTCTCTATGTAGGC |
|  | Reverse 5’- GCTCCTCCAACATTTGTCACTT |

**Supplementary Table S1 Related to qRT-PCR primer sequences**
