# Supplementary material for: Prognostic significance of SNCA and its methylation in bladder cancer
Source: BMC Cancer. 2022 Mar 26;22:330. doi: 10.1186/s12885-022-09411-9 (PMC8961938; doi:10.1186/s12885-022-09411-9)
Supplement: Supplementary file 8 — Additional file 8. [file 12885_2022_9411_MOESM8_ESM.docx]

**Supplementary table S8. Patients’ clinical characteristics information for tissue microarrays purchase form Outdo Biotech in this study.**

| Clinical characteristics | | Total  (63) | % |
| --- | --- | --- | --- |
| Age | >=65 | 41 | 66.1 |
|  | <65 | 21 | 33.9 |
| Gender | Male | 53 | 84.1 |
|  | Female | 10 | 15.9 |
| Stage | Ⅰ | 6 | 12.2 |
|  | Ⅱ | 11 | 22.4 |
|  | Ⅲ | 21 | 42.9 |
|  | Ⅳ | 8 | 16.3 |
|  | Ⅲ-Ⅳ | 3 | 6.2 |
| T stage | Tis | 5 | 8.6 |
|  | T1 | 11 | 19 |
|  | T2 | 14 | 24.1 |
|  | T3 | 25 | 43.1 |
|  | T4 | 3 | 5.2 |
| N stage | N0 | 45 | 84.9 |
|  | N1/N2 | 8 | 15.1 |
| M stage | M0 | 63 | 100 |
| CD8 | High | 32 | 50.8 |
|  | Low | 31 | 49.2 |
| PDL1 | High | 33 | 52.4 |
|  | Low | 30 | 47.6 |
