# Supplementary material for: Prognostic significance of SNCA and its methylation in bladder cancer
Source: BMC Cancer. 2022 Mar 26;22:330. doi: 10.1186/s12885-022-09411-9 (PMC8961938; doi:10.1186/s12885-022-09411-9)
Supplement: Supplementary file 9 — Additional file 9. [file 12885_2022_9411_MOESM9_ESM.pdf]

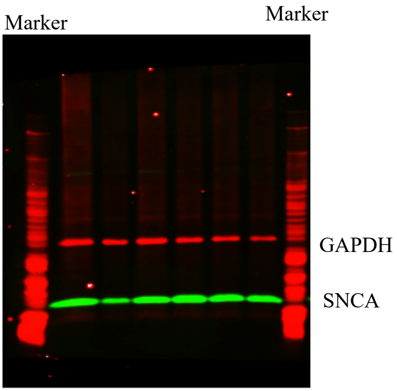

The specific detection for targart antigen in T24 Cells .GAPDH were labeled with goat anti mouse IRDye 680CW-conjugated antibody (red)and SNCA were labeled with goat anti rabbit IRDye 800CW-conjugated antibody(green).The blots bands were visualized using the Odyssey imaging system (Licor, USA).

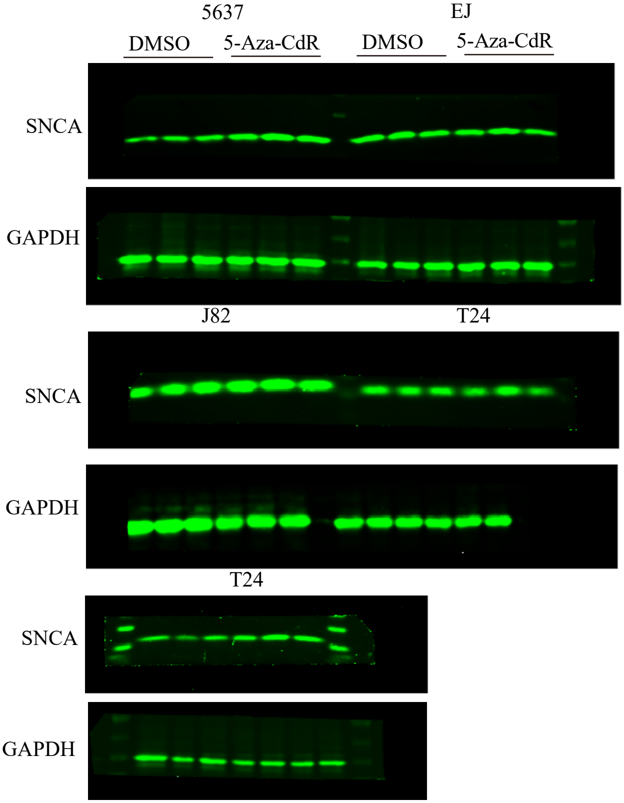

Supplementary Figure S3. The original images for Western blot in artical. The bands were visualized using the Odyssey imagging system(Licor, USA).
