# Supplementary figures and images for: Prognostic significance of SNCA and its methylation in bladder cancer
Source: BMC Cancer. 2022 Mar 26;22:330. doi: 10.1186/s12885-022-09411-9 (PMC8961938; doi:10.1186/s12885-022-09411-9)

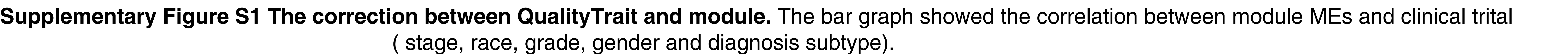

Supplement: Supplementary file 10 — Additional file 10. [file 12885_2022_9411_MOESM10_ESM.pdf]
