# Supplementary material for: Prognostic significance of SNCA and its methylation in bladder cancer
Source: BMC Cancer. 2022 Mar 26;22:330. doi: 10.1186/s12885-022-09411-9 (PMC8961938; doi:10.1186/s12885-022-09411-9)
Supplement: Supplementary file 11 — Additional file 11. [file 12885_2022_9411_MOESM11_ESM.pdf]

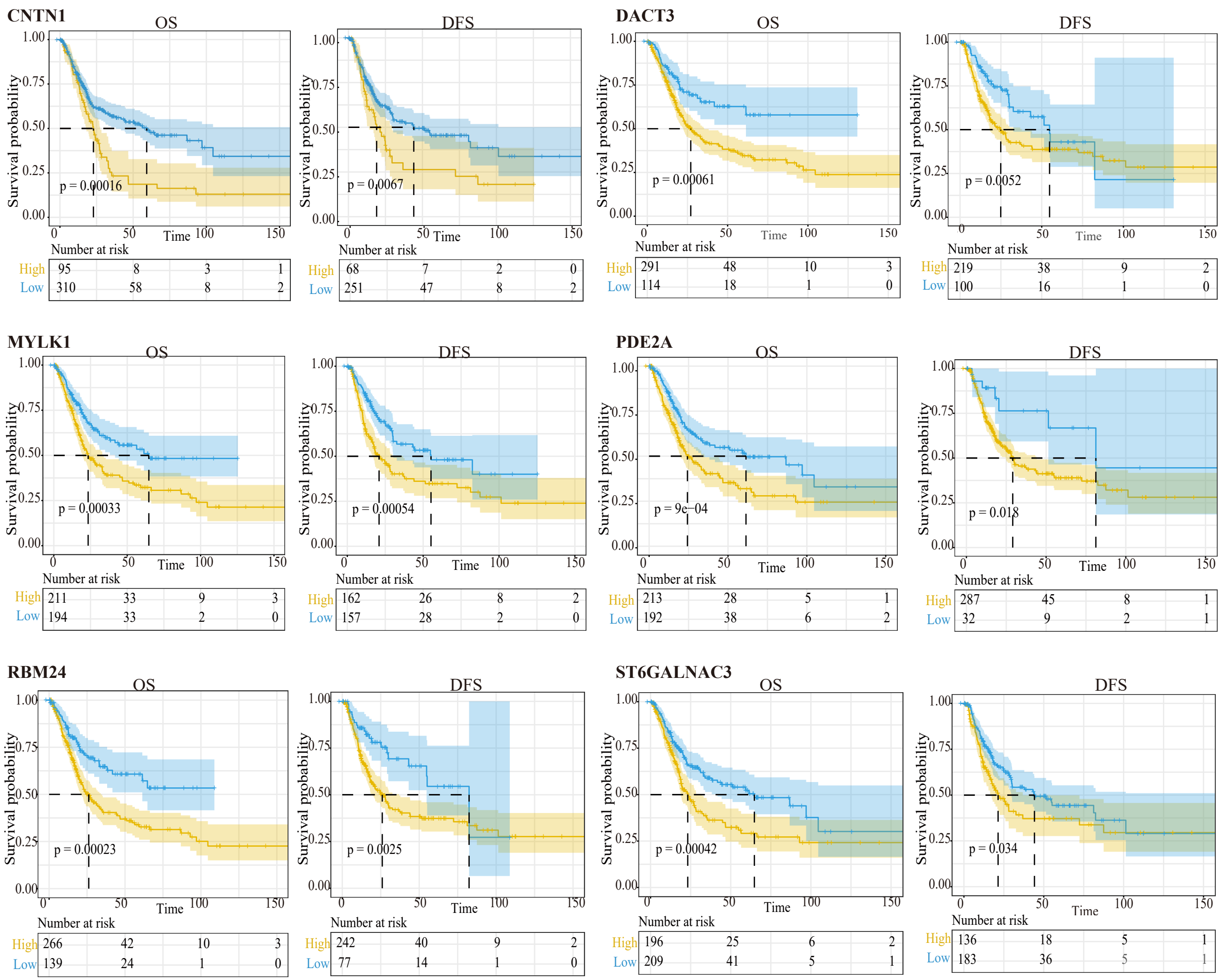

Supplementary Figure S2 Kaplan-Meier survival curves for OS and DFS of six key genes. The Log-Rank test was used to estimate OS and DFS differences between groups with different expression levels of SNCA. P -value < 0.05 was selected as threshold.
